# On the Effects of Transcranial Direct Current Stimulation on Cerebral Glucose Uptake During Walking: A Report of Three Patients With Multiple Sclerosis

**DOI:** 10.3389/fnhum.2022.833619

**Published:** 2022-01-25

**Authors:** Thorsten Rudroff, Alexandra C. Fietsam, Justin R. Deters, Craig D. Workman, Laura L. Boles Ponto

**Affiliations:** ^1^Department of Health and Human Physiology, University of Iowa, Iowa City, IA, United States; ^2^Department of Neurology, University of Iowa Health Clinics, Iowa City, IA, United States; ^3^Department of Radiology, University of Iowa Hospitals and Clinics, Iowa City, IA, United States

**Keywords:** multiple sclerosis, transcranial direct current stimulation, positron emission tomography, cerebral glucose uptake, caudate nucleus

## Abstract

Common symptoms of multiple sclerosis (MS) include motor impairments of the lower extremities, particularly gait disturbances. Loss of balance and muscle weakness, representing some peripheral effects, have been shown to influence these symptoms, however, the individual role of cortical and subcortical structures in the central nervous system is still to be understood. Assessing [^18^F]fluorodeoxyglucose (FDG) uptake in the CNS can assess brain activity and is directly associated with regional neuronal activity. One potential modality to increase cortical excitability and improve motor function in patients with MS (PwMS) is transcranial direct current stimulation (tDCS). However, tDCS group outcomes may not mirror individual subject responses, which impedes our knowledge of the pathophysiology and management of diseases like MS. Three PwMS randomly received both 3 mA tDCS and SHAM targeting the motor cortex (M1) that controls the more-affected leg for 20 min on separate days before walking on a treadmill. The radiotracer, FDG, was injected at minute two of the 20 min walk and the subjects underwent a Positron emission tomography (PET) scan immediately after the task. Differences in relative regional metabolism of areas under the tDCS anode and the basal ganglia were calculated and investigated. The results indicated diverse and individualized responses in regions under the anode and consistent increases in some basal ganglia areas (e.g., caudate nucleus). Thus, anodal tDCS targeting the M1 that controls the more-affected leg of PwMS might be capable of affecting remote subcortical regions and modulating the activity (motor, cognitive, and behavioral functions) of the circuitry connected to these regions.

## Introduction

Positron emission tomography (PET) imaging with the glucose analog [^18^F]fluorodeoxyglucose (FDG) can effectively measure cerebral glucose (the primary substrate used for ATP genesis) uptake and metabolism (Rudroff et al., 2020). Assessing FDG uptake approximates brain activity and is directly related to regional neuronal activity (Kennedy et al., 1975; Ginsberg et al., 1988; Niccolini et al., 2015). Because FDG remains localized in the brain during a 10–30 min uptake phase, it can map brain activity during tasks performed outside of the scanner (e.g., walking or running) (Tashiro et al., 2001). Thus, the tracer can be injected during a relevant activity and the resulting distribution of FDG can be measured after a known temporal delay. Using this methodology, FDG-PET images can represent a “brain metabolic signature” associated with physical performances (Rudroff et al., 2020).

Motor impairments, particularly in the legs, are frequent symptoms of multiple sclerosis (MS). Loss of balance and muscle weakness have been shown to influence these symptoms. However, the individual role of cortical and subcortical structures in the central nervous system is still to be understood. Applying FDG-PET at rest, Roelcke et al. (1997) and Bakshi et al. (1998) found reduced glucose metabolism within the brain of patients with MS (PwMS). Possible explanations for this disparity might be reduced brain volume and enlarged ventricles, which are common morphologies in PwMS (Grassiot et al., 2009), or altered glucose metabolism (Mathur et al., 2014). Bakshi et al. (1998) also indicated that cerebral dysfunction and neuronal system uncoupling might play an important role in the symptomatology of MS. In support of this, the results of Kindred et al. (2015) also suggested a decoupling of brain glucose utilization and motor task performance.

One potential modality to increase cortical excitability and improve motor function in PwMS is transcranial direct current stimulation (tDCS; Jeffery et al., 2007; Angius et al., 2016). tDCS is a non-invasive means of increasing neuronal excitability of regions under the anode (Nitsche and Paulus, 2000) and has been successfully used in healthy populations (Workman et al., 2019a, 2020a,b) and patients with neurological disorders like MS and Parkinson’s disease (PD; Fietsam et al., 2020; Workman et al., 2020c). Increasing the excitability of relevant brain regions might improve motor and cognitive function. Recent studies have shown that tDCS intensities ≤4 mA are safe, tolerable, and do not cause serious adverse effects (Bikson et al., 2016; Workman et al., 2019a; Khadka et al., 2020). However, the results of Workman et al. (2020d) agree with a critical review by Horvath et al. (2015) that questions the reliability of neurophysiological tDCS effects beyond the commonly reported alterations in motor evoked potential (MEP) amplitude. Specifically, Workman et al. (2020d) found no significant differences in regional or global cerebral blood flow [measured with (^15^O)water-PET] after 1, 2, 3, 4 mA, and SHAM stimulation. However, the authors did not assess the short-term effects of longer, more commonly used stimulation duration times (e.g., 20 min) and cannot exclude the possibility of long-term effects after 5 min of stimulation. Moreover, these results were found in a sample of PwMS with diverse disease severity and symptoms, which might contribute to the variability in tDCS responses. Thus, the lack of effect should be cautiously interpreted (Workman et al., 2020d).

Researchers have combined tDCS with other interventions, like physical (Kaski et al., 2014) and cognitive training (Agarwal et al., 2018; Dobbs et al., 2018). The notion driving such combinations is to promote greater synergistic effects than when the interventions are applied independently (Manenti et al., 2016; Broeder et al., 2019). In this framework, tDCS can either be applied concurrently (i.e., online) or as a priming technique (i.e., offline; before a performance). Such uses might strengthen long-term potentiation-like processes (Stoykov and Madhavan, 2015), supporting a greater maintenance of benefits from combined therapies (Costa-Ribeiro et al., 2016). Although tDCS combined with cognitive and physical training seems promising, the current results are mixed (Sánchez-Kuhn et al., 2017; Steinberg et al., 2018; Hsu et al., 2021). However, tDCS group results might not mirror individual subject responses (Wiethoff et al., 2014), which impedes our interpretation of the pathophysiology, treatment, and management of diseases like MS. Given the variabilities of both MS and tDCS, studies with a small number of patients reporting individual responses and highlighting commonalities could be useful for guiding hypotheses for future, larger studies. Thus, the purpose of this case report was to explore changes in cerebral glucose uptake after tDCS administered before a 20-min treadmill walk in three PwMS. The focus was not to investigate the effects of tDCS on motor function *per se*, but rather to assess individual neurological mechanisms relevant to the influence of tDCS on walking in PwMS.

## Methods

### Subject Criteria

All three subjects met the following inclusion criteria: (1) 18–70 years old, (2) relapsing-remitting MS (revised McDonald’s criteria) (Thompson et al., 2018), (3) able to walk for 20 min without rest, and (4) self-reported deficit in unilateral leg strength. Exclusion criteria were: (1) unable to fast for 6 h, (2) pregnancy, (3) history of seizures or taking medication known to lower the seizure threshold, (4) modifications to disease-altering medication in the last 45 days, (5) MS relapse within the last 60 days, (6) high risk for cardiovascular disease under the American College of Sports Medicine risk classification system (Thompson et al., 2013), (7) concurrent neurological or neuromuscular disorder, (8) depression, (9) inability to understand and sign the consent form, and (10) hospitalization within the last 90 days. The study was approved by the Institutional Review Board at the University of Iowa and conducted per the Declaration of Helsinki (i.e., subjects signed informed consent before participating).

### Study Protocol

**FIGURE 1:**
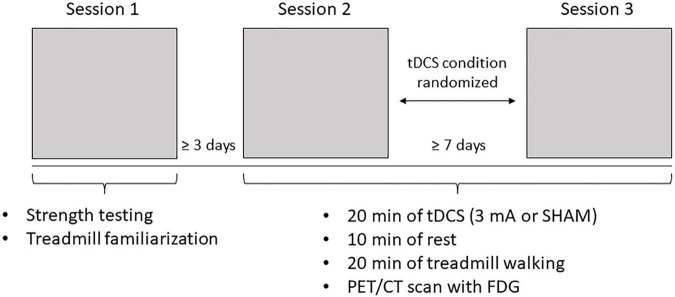
Experimental protocol. Subjects attended three sessions. Sessions 1 and 2 were spaced at least 3 days apart and Sessions 2 and 3 were spaced at least 7 days apart. During Session 1, subjects completed strength testing to objectively determine their more-affected leg and treadmill familiarization. During Sessions 2 and 3, subjects underwent 20 inin of either Sham or 3 mA Ml tDCS (determined through randomization), followed by 20 inin of treadmill walking after a 10 inin rest, then a whole body positron emission tomography/computer tomography (PET/CT) scan with fluorodcoxyglucosc (FDG).

This study employed a single-blind, SHAM-controlled, cross-over design. The subjects attended three sessions with ≥3 days between Sessions 1 and 2 (to allow adequate leg muscle recovery from the isokinetic strength test, detailed below) and ≥7 days between Sessions 2 and 3 to ensure the effects of tDCS were sufficiently diminished (Nitsche and Paulus, 2000; Fietsam et al., 2020, 2021; Figure 1). During Session 1, the subjects provided informed consent and completed the Patient Determined Disease Scale (PDDS) and Fatigue Severity Scale (FSS). The PDDS is a self-reported scale that probes subjective disability and is strongly correlated with the clinician-administered Expanded Disability Status Scale (EDSS; Learmonth et al., 2013). PDDS scores range from 0 (normal) to 8 (bedridden), with higher scores indicating greater disability. The FSS measures self-reported fatigue and averages patient responses to nine items scored on a 0 to 7 scale (higher scores indicate greater fatigue). After completion of the questionnaires, isokinetic strength testing (see below) was performed to objectively determine the more-affected leg; if the difference between the legs was <10%, the subject’s self-reported weaker leg was deemed more affected. After strength testing, the subjects self-selected a comfortable walking speed, which was used for the treadmill task in Sessions 2 and 3. The subjects fasted for ≥6 h before these sessions. Upon arrival, their blood glucose levels, height, and weight were measured, and an IV was inserted to facilitate FDG administration. Blood glucose needed to be <200 mg/dL to proceed with FDG administration/scanning. The subjects then sat in a chair and tDCS was administered [active (3 mA) or SHAM; randomized] for 20 min. The anode was placed over the motor cortex representation corresponding with the more-affected leg, and the cathode was placed contralateral to the anode on the supraorbital area. Following stimulation, the subjects rested for 10 min to facilitate peak tDCS effects (Jeffery et al., 2007; Santarnecchi et al., 2014). After the rest period, subjects walked on the treadmill at the self-selected speed from Session 1.2 min into the 20-min treadmill walking task, the FDG tracer (∼10 mCi) was injected. Promptly after the treadmill task, subjects were led to the PET/CT scanner and a whole body (head to toes) scan was performed.

### Isokinetic Strength Testing

Strength testing was performed on an isokinetic dynamometer (HUMAC NORM, CSMi, Stoughton, MA, United States). Session 1 commenced with a 15-repetition warmup (60°/s, concentric/concentric) of the knee extensors and flexors followed by ≥30 s rest. The strength protocol included maximal isokinetic knee extension and flexion (concentric/concentric; 60°/s; five sets of one rep with ≥30 s between sets). Maximum torque on each leg was used to objectively determine the more-affected leg (Workman et al., 2019a,b). Strength testing was always performed on the right leg followed by the left leg with ≥2 min of rest between the legs. Vigorous verbal encouragement was provided to encourage a maximum effort from each repetition (Workman et al., 2019a,b).

### Transcranial Direct Current Stimulation

Stimulation was delivered from a handheld device (Soterix Medical Inc., New York, NY, United States) through two carbon electrodes placed inside saline-soaked sponges (5 cm × 7 cm). The anode was centered over the motor cortex (M1) of more-affected leg (either C3 or C4) and angled at 45° relative to the coronal plane (Foerster et al., 2019). The size of the anode ensured that the center (Cz) of the skull was slightly covered and the leg area of the primary motor cortex (in the longitudinal fissure) was unilaterally targeted (Jayaram and Stinear, 2009; Foerster et al., 2018; Fietsam et al., 2020). The cathode was placed over the contralateral supraorbital area. During active stimulation, the current ramped up from 0 mA to 3 mA over 30 s, then remained at 3 mA for 20 min before a 30 s ramp down to 0 mA. During SHAM, the stimulation ramped up to 3 mA over 30 s then immediately ramped down from to 0 mA over 30 s during the first and last minute of the 20-min period; the stimulation otherwise remained at 0 mA. SHAM stimulation produces similar sensations as active tDCS and is used to maintain condition blinding without altering cortical excitability (Woods et al., 2016). These tDCS parameters have been used previously in our lab (Fietsam et al., 2020, 2021). Following tDCS, the subjects were asked which condition (3 mA or SHAM) they thought they received and described the sensations felt during the stimulation, rated on a 1–10 Likert scale (1 = “barely perceptible; 10 = “Most I could possibly stand”). Responses were recorded, but blinding integrity was maintained until the subject completed all study sessions.

### Positron Emission Tomography Image Acquisition

Positron emission tomography/CT scans were acquired using a GE Discovery MI Time of Flight PET/CT scanner with SiPM array detector technology (GE Healthcare, Waukesha, WI, United States). CT scans were obtained first to allow for attenuation correction. The subjects were strapped in place on the scanning table to minimize movement during acquisition. A 3-dimensional row-action maximum likelihood algorithm (RAMLA; 2 × 2 × 2 mm voxel) method was utilized to reconstruct attenuation-corrected images.

### Positron Emission Tomography Image and Regional Analysis

All images were analyzed with PNEURO (PMOD 4.0; PMOD Technologies LLC, Zurich, Switzerland). In this process, 78 volumes of interest (VOIs) were estimated using the Hammers N30R83 maximum probability atlas and then were spatially normalized in Montreal Neurological Institute space using a normal brain template. For each VOI, standardized uptake values (SUVs) were calculated and normalized to body weight. A volume-weighted global mean uptake was calculated and relative regional metabolism (i.e., normalized to the global mean value) was calculated for each region. To compare differences in metabolism between active and SHAM conditions, the relative regional metabolism in the SHAM condition was subtracted from the relative regional metabolism for the active condition, divided by the relative regional metabolism for the active condition, and multiplied by 100 to give a percent difference between active/SHAM for a specific region (e.g., {[right caudate nucleus (active)] – [right caudate nucleus (SHAM)]/right caudate nucleus (active)} × 100) (Fietsam et al., 2020). The brain regions of interest included M1 and the surrounding areas (regions under the anode), the orbital gyri directly (regions under the cathode), and the basal ganglia, which are part of relevant motor performance circuits (Simonyan, 2019).

## Results

### Transcranial Direct Current Stimulation Sensations and Blinding

All of the subjects completed all sessions, and no data were missing. Two subjects correctly guessed r SHAM, and one correctly guessed active stimulation. The most common sensations during the active condition were burning (5.5 ± 3.54), headache (3.0 ± 0), and tingling (2.0 ± 0). The most frequently reported sensations during the SHAM condition were burning (7.0 ± 0), pins and needles (6 ± 1.41), and headache (5 ± 0).

### Subject 1

#### Demographics/Pre-testing

Subject 1 was a 27-year-old male, with a height, weight, and BMI of 168 cm, 81 kg, and 28.7 kg/m^2^, respectively. Subject 1 had been diagnosed with MS for 12 years, reported a score of 0 on the PDDS, a score of 1.78 on the FSS, and was not physically active according to the stated guidelines. Peak torque was greater for the left knee extensors than the right extensors (127 Nm vs. 98 Nm). Hence, the anode was placed over the left M1 (C3) corresponding to his right, more-affected leg. The treadmill was set to 2.7 mph, and blood glucose levels were 90 mg/dL (Session 2; SHAM) and 85 mg/dL (Session 3; active).

#### Relative Regional Metabolism Changes

In Subject 1, the areas under the anode and cathode (lt middle frontal gyrus, lt precentral gyrus, lt superior frontal gyrus, rt anterior orbital gyrus, rt medial orbital gyrus, rt lateral orbital gyrus) had minimal changes in uptake (all within +2.5% or −2%) after 3 mA stimulation compared with SHAM. However, in the basal ganglia (rt/lt caudate nucleus, rt/lt nucleus accumbens, rt/lt putamen, rt/lt thalamus, rt/lt pallidum, rt/lt substantia nigra), more regions showed increased uptake (Table 1), most notably the lt pallidum (4.48%) and rt caudate nucleus (3.39%).

**TABLE 1:** Relative regional metabolism in motor areas beneath the anode, *cathode* and the basal ganglia.

	SUBJECT 1			SUBJECT 2			SUBJECT 3		
REGION	Active	Sham	Difference	Active	Sham	Difference	Active	Sham	Difference
Lt middle frontal gyrus	1.22	1.23	−0.87%	1.28	1.29	−0.69%	1.33	1.30	2.29%
Lt precentral gyrus	1.15	1.15	0.41%	1.18	1.18	−0.18%	1.15	1.13	1.24%
Lt superior frontal gyrus	1.10	1.09	0.99%	1.14	1.12	1.89%	1.13	1.11	1.94%
*Rt anterior orbital gyrus*	1.174	1.191	−1.49%	1.186	1.228	−3.49%	1.222	1.175	3.91%
*Rt medial orbital gyrus*	1.128	1.116	1.02%	1.192	1.175	1.44%	1.159	1.094	5.64%
*Rt lateral orbital gyrus*	1.226	1.195	2.51%	1.377	1.409	−2.33%	1.258	1.214	3.50%
**Lt caudate nucleus**	0.95	0.93	1.58%	0.78	0.74	5.43%	0.92	0.93	−2.05%
**Rt caudate nucleus**	0.82	0.79	3.39%	0.72	0.67	6.16%	0.87	0.80	8.68%
**Lt nucleus accumbens**	1.12	1.15	−2.66%	1.08	1.06	1.65%	1.30	1.27	2.65%
**Rt nucleus accumbens**	0.94	0.93	0.78%	0.91	0.93	−2.78%	1.04	1.02	2.63%
**Lt putamen**	1.28	1.27	1.03%	1.25	1.21	3.06%	1.42	1.36	4.41%
**Rt putamen**	1.22	1.20	1.60%	1.25	1.22	2.58%	1.40	1.34	4.30%
**Lt thalamus**	1.01	1.03	−1.97%	0.88	0.86	1.95%	1.09	1.07	2.17%
**Rt thalamus**	1.03	1.04	−0.88%	0.93	0.90	3.98%	1.06	1.06	0.07%
**Lt pallidum**	1.02	0.97	4.48%	0.97	0.93	4.67%	1.12	1.09	2.26%
**Rt pallidum**	0.77	0.77	0.36%	0.82	0.84	−2.46%	0.95	0.93	1.38%
**Lt substantia nigra**	0.87	0.90	−2.40%	0.96	0.92	3.70%	0.91	0.90	4.28%
**Rt substantia nigra**	0.97	0.99	−2.13%	0.95	0.98	-3.21%	1.07	0.98	−7.38%

Right caudate nucleus differences [(active – sham)/active]*100 are shown in red.

### Subject 2

#### Demographics/Pre-testing

Subject 2 was a 44-year-old female with a height, weight, and BMI of 155 cm, 62 kg, and 25.81 kg/m^2^, respectively. This subject had been diagnosed with MS for 14 years, scored a 3 on the PDDS, a 7 on the FSS, and was physically active. The left knee extensors had the highest torque (57 Nm vs. 48 Nm). Therefore, the anode was placed over the left M1 (C3). The treadmill speed was 2.0 mph and blood glucose was 81 mg/dL (Session 2; SHAM) and 83 mg/dL (Session 3; active).

#### Relative Regional Metabolism Changes

In Subject 2, the areas under the anode showed a similar activity pattern as Subject 1, with slightly decreased activity in the lt middle frontal and lt precentral gyrus and a small (1.89%) increase in the lt superior frontal gyrus during active compared with SHAM (Table 1). The areas underneath the cathode displayed conflicting results from Subject 1 with decreased activity in the anterior and lateral rt orbital gyri (−3.49; −2.33%, respectively) and a slight increase (1.44%) in the rt medial orbital gyrus. In the basal ganglia, two-thirds of the regions displayed increased activity, particularly the rt caudate nucleus (6.16%), lt caudate nucleus (5.43%), and lt pallidum (4.67%).

### Subject 3

#### Demographics/Pre-testing

Subject 3 was a 57-year-old female with a height, weight, and BMI of 165 cm, 51 kg, and 18.7 kg/m^2^, respectively. Her time since diagnosis was 32 years. She reported a 3 on the PDDS, a score of 4.2 on the FSS, and was physically active. The left knee extensors had a higher torque than the right knee extensors (60 Nm vs. 36 Nm). Hence, the anode was placed over the left M1 (C3). The treadmill speed was 0.8 mph and her blood glucose was 110 mg/dL (Session 2; active) and 109 mg/dL (Session 3; sham).

#### Relative Regional Metabolism Changes

**FIGURE 2:**
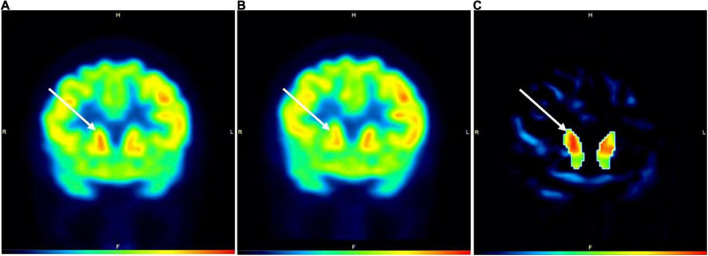
PET images during active **(A)** and SHAM **(B)** conditions in Subject3. Image **(C)** represents a subtraction PET image with the globally normalized activity in sham subtracted from the globally normalized activity in active. All areas in image **(C)** are masked except for the caudate nuclei. White arrows denote the location of the caudate nuclei. The color bar indicates level of increased FDG uptake (black = no uptake; red = highest glucose uptake). H = head; F = foot; R = right; L = left.

In Subject 3, the areas under the anode and cathode showed a modest increase in activity (1.94% – 5.64%; Table 1) in active compared to SHAM, with all areas under the cathode showing at least 3.5% increases in activity. For the basal ganglia, the rt caudate nucleus displayed an 8.68% increase, the lt substantia nigra, rt putamen, and lt putamen all had >4% increase in activity, and the rt substantia nigra was the only area that showed a decrease in activity (−7.38%) in active compared to SHAM (Table 1 and Figure 2).

## Discussion

This case report indicated that tDCS over the left M1 was capable of affecting remote, subcortical regions, like the caudate nucleus, in PwMS during walking. Thus, tDCS might be a useful treatment opportunity for patients with progressive basal ganglia degeneration, such as multiple sclerosis and PD (Haussleiter et al., 2009; Draoui et al., 2020). The caudate nuclei are paired nuclei. In addition to the globus pallidus and putamen (corpus striatum), they make up the basal ganglia (Villablanca, 2010). The caudate nuclei have both behavioral and motor functions, including body and limb posture and controlling approach-attachment behaviors, respectively (Kalron et al., 2020). Of note, the basal ganglia have strong anatomical and functional connections with the cortex, thalamus, and brainstem, and play a relevant role in initiating and controlling locomotion (Villablanca, 2010; Onu et al., 2015; Kalron et al., 2018, 2020). Previous studies examining the association between magnetic resonance imaging (MRI) measures and gait in PwMS have found that gray matter volume of the putamen, caudate, globus pallidus, and nucleus accumbens were all linked to the Timed 25-ft Walk (T25FW) test in relapsing-remitting MS (Alexander et al., 1990; Onu et al., 2015), whereas cerebellar volume was associated with the T25FW test in primary progressive MS (Alexander et al., 1990; Bhatia and Marsden, 1994). Moreover, Kalron et al. (2018) reported that PwMS with a history of falls exhibited reduced left caudate volume compared to non-fallers.

fluorodeoxyglucose-PET studies in PwMS have reported reduced cerebral glucose metabolism in the frontal cortex and basal ganglia (Roelcke et al., 1997; Bakshi et al., 1998). Lesions of the dorsolateral prefrontal cortex were also characterized by deficits in motor programming and executive function, evidenced in alternating and sequential tasks, as well as the vacillating task demands of tests like the Wisconsin Card Sorting Test (Alexander et al., 1990). Furthermore, lesions of the caudate nucleus frequently result in low initiative, motivation, and poor task maintenance (Alexander et al., 1990; Bhatia and Marsden, 1994). This might indicate that disseminated areas of demyelination could result in reductions of glucose metabolism in cortical or subcortical regions. Thus, motor impairments might be ascribed to a disruption of distinct cortico-subcortical circuits. Among these, the dorsolateral prefrontal circuit connects the dorsolateral prefrontal cortex to the lateral head of the caudate nucleus (Alexander et al., 1990). Additionally, the motor circuit originates from the supplementary motor area, premotor, and sensorimotor areas of the frontal cortex and links these regions to the putamen (Alexander et al., 1990). Both circuits project *via* other basal ganglia nuclei to the thalamus and then back to the frontal cortex (Simonyan, 2019). Based on these studies, it is suggested that tDCS applied to the M1 might modulate neural activity of either circuit, which would mediate motor, cognitive, and behavioral function.

Studies in non-MS populations have also shown the association of the caudate nucleus with the maintenance of balance and walking velocity, such as patients with progressive supranuclear palsy (Villablanca, 2010; Kalron et al., 2018). In particular, the findings of Palmisano et al. (2020) indicated a major role of the caudate nucleus in the planning and execution of the gait initiation program. Also, patients with PD and postural instability exhibited caudate atrophy and decreased functional connectivity between the caudate nucleus and supplementary motor area (Jacobs et al., 2009), which is directly involved in producing anticipatory postural adjustments (MacKinnon et al., 2007; Arnulfo et al., 2018). Furthermore, volume change in the caudate nucleus has been found in PD (Zhang et al., 2016; Pasquini et al., 2019). Patients with early caudate denervation in PD, as measured by 123I-FP-CIT SPECT, were associated with more significant gait impairment at a four-year follow-up (Dumurgier et al., 2012). Additionally, compared with the tremor-dominant PD subtype, the postural instability and gait disturbance (PIGD) subtype had metabolic reductions in the caudate (Pasquini et al., 2019). The results of Zhang et al. (2016) revealed associations between caudate microstructural changes with cadence and stride time in PD subjects OFF medication. The authors further stated that these correlations might help elucidate the neuropathological basis of gait impairment in PD (Zhang et al., 2016), which agrees with the finding that caudate changes were related to dopaminergic neuronal loss (Pozzi et al., 2019). Lastly, in healthy older adults (>65 years), caudate atrophy was related to gait speed (Dumurgier et al., 2012) and striatal dopamine denervation in the caudate, as measured by [11C]-β-CFT dopamine transporter PET imaging, was related to reductions in cadence and gait speed (Bhatia and Marsden, 1994). Considered together, these results highlight the important role of the caudate nucleus in functional gait in a variety of populations.

In addition to the caudate nucleus, our results indicated increased glucose uptake in the left pallidum after 3 mA tDCS. Using fMRI, Motl et al. (2015) found that the thalamus and basal ganglia nuclei, particularly the pallidum and caudate, were associated with walking outcomes (i.e., 6-Min Walk Test and Timed 25-Foot Walk Test) in PwMS. The authors hypothesized that the pallidum was the strongest predictor of performance because it represents a common relay for cortico-subcortical loops involved in motor function in PwMS.

Lastly, our findings expand on the results of Polania et al. (2012), who hypothesized that M1 tDCS would result in different functional connectivity between striatal and thalamic regions and motor-related cortical regions. They found that 10 min of 1 mA anodal tDCS inside an MRI scanner enhanced the connectivity between the left M1 and the ipsilateral thalamus, superior parietal lobule, and left caudate nucleus, and decreased the connectivity between the caudate nucleus and the left posterior cingulate cortex. However, they used resting-state fMRI, whereas the current study used FDG-PET to investigate the modulation of brain activity during treadmill walking after tDCS administered before the task (i.e., as a primer).

### Study Limitations

We used a bipolar cephalic electrode montage (anodal and cathodal electrodes with equivalent sizes). It can be considered that the excitability enhancement of the frontal cortex *via* anodal stimulation-induced functional activity changes in connected ipsilateral subcortical structures, like the thalamus and putamen. This, together with the cathodal-induced excitability reduction of the contralateral frontopolar cortex, might result in desynchronization of these areas. In other words, because both electrodes were located on the head, the contribution of each electrode to the resultant brain activity changes cannot be distinguished. However, this problem might be overcome in future studies by using extracephalic montages (Workman et al., 2020c) or by increasing the size of the reference electrode to make it functionally inert (Bhatia and Marsden, 1994). The low temporal resolution of FDG-PET is another limitation. Particularly, levels of brain activity may not have been stable (i.e., fluctuated) during walking and this might not have been measurable with this imaging technique. Additionally, the small sample size of people with relapsing-remitting MS is a limitation of the study and limits the generalizability of the results.

### Summary and Future Studies

This case series found that anodal tDCS targeting the M1 that controls the more-affected leg of PwMS might affect remote subcortical regions, like the caudate nucleus, in PwMS. This suggests that M1 tDCS might increase neural activity of the dorsolateral prefrontal and/or motor circuits, which would mediate motor, cognitive, and behavioral functions within the brain. In the future, more specific evaluations of the impact of fatigue, depression, anxiety, pain, and MS medications on cortical function alterations should be employed. Furthermore, future studies should link tDCS with task-related paradigms to investigate the mechanisms of stimulation-induced functional cortico-subcortical and cortico-cortical modulations. These and other recent findings provide evidence that tDCS can generate alterations in brain activity beyond the stimulation site, supplemented by changes in glucose metabolism, cerebral blood flow, and neurotransmitters. However, the applied current intensity/density, stimulation duration, behavioral paradigm, and state of the subjects (i.e., resting or during a motor task) varied in these studies. Consequently, systematic manipulation and investigation, of these relevant stimulation parameters in future studies will be crucial.

## Data Availability Statement

The original contributions presented in the study are included in the article/supplementary material, further inquiries can be directed to the corresponding author.

## Ethics Statement

The studies involving human participants were reviewed and approved by Institutional Review Board at the University of Iowa. The patients/participants provided their written informed consent to participate in this study.

## Author Contributions

All authors have participated in the procurement of this manuscript and agreed with the submitted case report.

## Conflict of Interest

The authors declare that the research was conducted in the absence of any commercial or financial relationships that could be construed as a potential conflict of interest.

## Publisher’s Note

All claims expressed in this article are solely those of the authors and do not necessarily represent those of their affiliated organizations, or those of the publisher, the editors and the reviewers. Any product that may be evaluated in this article, or claim that may be made by its manufacturer, is not guaranteed or endorsed by the publisher.
